# Unexpected Adverse Effect of Haloperidol: Acute Tongue Angioedema in a Schizophrenic Patient—Case Report and Review

**DOI:** 10.1155/crps/4133014

**Published:** 2025-06-24

**Authors:** Nadia Romdhane, Dorra Chiboub, Ameni Amri, Asma Ayedi, Emna Rejeb, Imen Zoghlami, Safa Nefzaoui, Ines Hariga, Chiraz Mbarek

**Affiliations:** ^1^Ears Nose and Throat, Head and Neck Surgery Department, Habib Thameur Hospital, Tunis, Tunisia; ^2^Faculty of Medicine of Tunis, University Tunis El Manar, Tunis, Tunisia

**Keywords:** airway obstruction, antipsychotic agents, drugs, edema

## Abstract

Angioedema of the tongue, also known as angioneurotic, or Quinke edema is a swelling of the tongue due to plasma leaking from capillary and postcapillary venules into deep submucosal tissue. This condition can either be hereditary, or acquired, due to allergy induced reactions for example. With an acute onset, this phenomenon can potentially be life threatening due to sudden and complete upper airway obstruction. Our aim is to describe the case of a 54-year-old schizophrenic male patient who presented with an angioedema of the tongue occurring after oral administration of haloperidol, a first-generation antipsychotic. The patient was admitted for close respiratory monitoring. The established cause for this condition was an allergic reaction to haloperidol. The following course was a favorable outcome with complete resolution of the edema without respiratory distress. We aim to report our case and to delve into other existing similar cases reported thus far in literature.

## 1. Introduction

Haloperidol is a first-generation typical antipsychotic medicine that primarily inhibits dopamine D2 receptors of the brain. It is largely used to treat schizophrenia positive symptoms and a wide array of psychotic disorders. Despite the efficiency of this drug, a variety of adverse effects have been reported. The main side effects are of neurological order, with the most common ones being extrapyramidal symptoms (EPSs) and sedation. Angioedema is a rare however potentially deadly adverse response, characterized by localized deep swelling caused by increased vascular permeability and blood flowing from vessels into submucosal tissue. The term “Quinke edema” is often associated with angioedema of the uvula, but it can also refer to angioedema affecting other parts of the upper airway, including the tongue. In this case, the patient presented with angioedema of the tongue without laryngeal involvement, as confirmed by flexible nasofibroscopy, which showed an open airway and no laryngeal edema. The term “angioedema” is used here to describe the localized swelling of the tongue due to increased vascular permeability, consistent with the pathophysiology of angioedema. Symptoms and prognosis of this condition depend directly on the location of the swelling. When involving the larynx or the oro-lingual region, this swelling can lead to acute upper airway obstruction with a sudden onset of dyspnea. Acute angioedema of the tongue due to haloperidol administration is an extremely rare adverse effect of this antipsychotic, with only four cases reported in the literature. Our aim is to report a rare case of angioedema of the tongue occurring immediately after oral haloperidol administration, highlighting the clinical presentation and its course, as well as to provide a comprehensive review of the existing literature on similar cases that have been reported.

## 2. Material and Methods

We studied the case of a 54-year-old male patient who presented with an acute angioedema of the tongue following haloperidol administration.

## 3. Case Report

**Figure 1:**
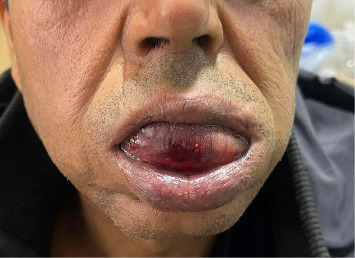
Protrusion and swelling of the tongue.

**Figure 2:**
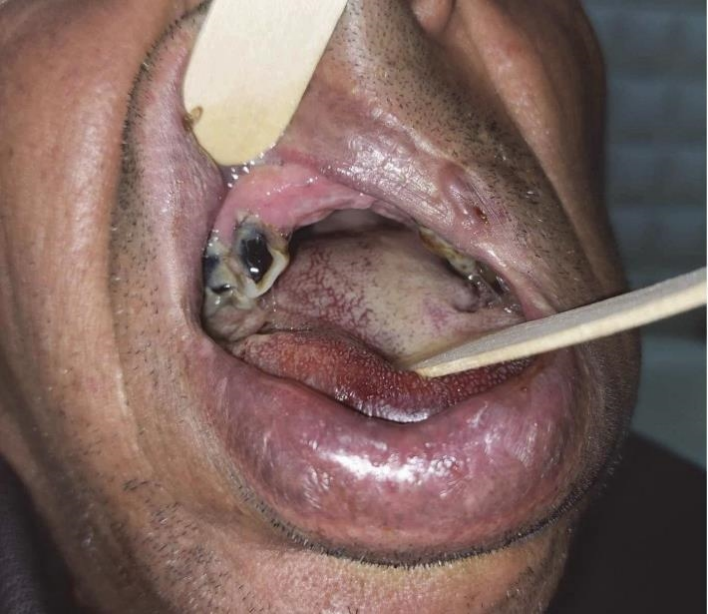
Bilateral and posterior extension of tongue swelling.

**Figure 3:**
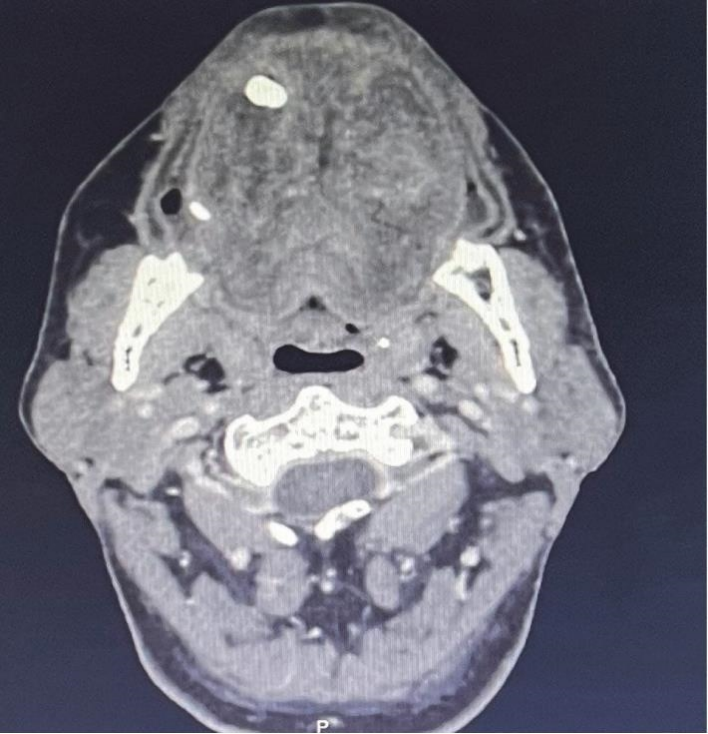
Axial CT scan of the oropharynx revealing heterogenous swelling of the tongue.

A 54-year-old male patient was referred to the emergency department of otolaryngology for an acute swelling of the tongue. He had a history of hepatitis B virus infection and a diagnosis of late-onset schizophrenia for 1 year, for which he was prescribed haloperidol, biperiden, and lorazepam. The patient had already been on haloperidol for 1 year as part of his treatment for late-onset schizophrenia. There was no history of drug allergy. The patient's symptoms appeared about 20 min after an oral administration of two haloperidol pills. The family reports no epileptic or trauma events. A prompt and rapidly increasing tongue swelling occurred, along with pain and discomfort from jaw clenching. The patient reported difficulties with speech and feeding without signs of dyspnea. On clinical examination, vital signs were reassuring. The patient was afebrile, eupneic with normal ambient air saturation. There were no signs of confusion, memory impairment, or cognitive deficits. All cranial nerves were intact. There was no sensory nor motor function impairment besides oromandibular dystonia. In fact, the patient presented with a clenched jaw, which made examination of the oropharynx difficult. There was additionally protrusion of the tongue, which was edematous, associated with hypersalivation (Figure 1). The tongue edema involved both free edges, extending bilaterally and symmetrically from the tip to the dorsal surface, without evidence of biting or trauma (Figure 2). Flexible nasofibroscopy revealed an open airway, with no laryngeal edema. The patient was admitted to our unit for close respiratory monitoring. After concertation with the psychiatrists, it was decided to stop his long-term antipsychotic treatment. The immediate laboratory workup showed correct hemostasis, with neutrophil-dominated hyperleukocytosis at 29,000 and c-reactive protein (CRP) at 109 mg/L. An emergency cervicofacial computed tomography (CT) scan showed a swollen and edematous aspect of the tongue, protruding anteriorly and laterally, with heterogeneous enhancement and no collection or hematoma (Figure 3). The diagnosis of an allergic reaction to haloperidol in the form of angioedema of the tongue associated with oromandibular dystonia was established. Alternative diagnoses, such as isolated oromandibular dystonia or other neurological reactions, were considered but ruled out based on the clinical presentation. The patient's symptoms of acute tongue swelling, hypersalivation, and jaw clenching were consistent with angioedema rather than a purely dystonic reaction. Additionally, the absence of other EPS and the rapid onset of swelling following haloperidol administration supported the diagnosis of angioedema. Imaging also confirmed the absence of hematoma, purulent collection, or trauma, further ruling out other causes of tongue swelling. Given the feeding difficulties, the patient was given an enteral diet. In view of the local conditions, the risk of superinfection and the biological inflammatory syndrome, antibiotic therapy based on third-generation cephalosporin with metronidazole was instituted. The rationale for antibiotic use in this case was prophylactic, given the elevated inflammatory markers (CRP at 109 mg/L and neutrophil-dominated hyperleukocytosis at 29,000), and the risk of superinfection due to the compromised oral mucosa and feeding difficulties. Although no confirmed infection was present, the inflammatory response and the potential for secondary infection in the context of tongue swelling and oromandibular dystonia justified the use of antibiotics. The antibiotic regimen included third-generation cephalosporin with metronidazole to cover both aerobic and anaerobic bacteria, which are common in oropharyngeal infections. The inflammatory markers were likely elevated due to the acute allergic reaction and the associated tissue swelling. The absence of fever, localized infection, or other signs of systemic infection ruled out an infectious cause. The decision to use antibiotics was purely prophylactic, given the risk of superinfection in the context of compromised oral mucosa and feeding difficulties. The edema and dystonia began to resolve on the third day of treatment, with complete disappearance on the fifth day and restoration of normal eating and speech abilities. The patient did not receive specific allergy medication such as antihistamines or corticosteroids, as the primary management focused on discontinuing haloperidol, providing supportive care, and monitoring for respiratory compromise. The swelling and dystonia began to resolve on the third day of treatment, with complete resolution by the fifth day, suggesting that the removal of the offending agent (haloperidol) was sufficient for recovery. The biological inflammatory syndrome had a descending curve from the second day of treatment, with negativation within 5 days. Given the regression of clinico-biological signs, and the absence of respiratory and neurological deterioration, the patient was discharged after 1 week of treatment. The 2-week follow-up noted a tongue of normal size and mobility, a regular mouth opening, and a healthy oropharynx.

## 4. Discussion

Angioedema is defined as an acute nonpitting swelling of subcutaneous and submucosal tissues [1]. This can occur in any part of the body with the most frequent regions involving the face, lips, tongue, and throat. However, it can also occur in the gastrointestinal tract, genitals, and extremities [2]. Vital prognosis depends sharply on location of the swelling, making this condition potentially lethal when involving in the head and neck region as it can abruptly obstruct airway patency [3]. The risk of this complication implies a dynamic and prompt management, which requires an established or at least suspected diagnosis based on the clinical presentation [4]. In fact, angioedema can either be acquired or genetic. Hereditary angioedema is a rare genetic disorder mostly related to the C1-esterase inhibitor protein [5]. This condition is either due to decreased amounts of C1-INH protein (type 1) or decreased function of C1-INH (type 2). Apart from these cases, hereditary angioedema is explained by other genetical defects affecting factor XII, angiopoietin-1, plasminogen, and kininogen 1 genes [6]. Hereditary angioedema is an autosomal dominant condition manifesting as recurrent episodes of swelling. Treatment plan involves long-term prophylactic injections of C1-INH enzyme replacement. Management of acute onset episodes relies primarily on intravenous infusion of plasma and recombinant C1-INH. When assessing a patient presenting a first episode of angioedema with no similar family history, it is key to keep in mind that this condition can be acquired, triggered by a broad spectrum of external factors. To differentiate between both causes, it is useful to determine the blood concentration of C1q, which is low in acquired angioedema, unlike hereditary cases [7]. Most common external factors of angioedema include food allergy, a histamine-mediated immune response to certain proteins manifesting in any part of the body. Oral allergy syndrome may involve angioedema of the tongue as an immediate allergic reaction to raw fruits and vegetables having common proteins with pollen [8]. Additionally, angioedema of the tongue can also develop as a systemic reaction to insect bites. Bee or wasp stings can provoke an immediate and systemic response triggered by the venom that induces a histamine-mediated extensive swelling which may lead to anaphylaxis [9]. Treatment in histamine-induced angioedema of the tongue depends on severity of symptoms. When assessing these cases, it is crucial to rule out and manage respiratory distress and circulatory collapse. Treatment plan consists of antihistaminic drugs, steroids, and even epinephrine in cases with distress [10]. Another cause of histamine-induced angioedema is drugs. Beta-lactam antibiotics, such as penicillin and cephalosporins, are known to provoke histamine release and hypersensitivity reactions, which can lead to angioedema. These medications often trigger an immune response that results in the degranulation of mast cells and the subsequent release of histamine, a key mediator in the development of angioedema. Additionally, angiotensin converting enzyme (ACE) inhibitors are another group of drugs that can induce angioedema. The mechanism here involves the accumulation of bradykinin, a peptide that promotes vasodilation and increases vascular permeability. Elevated bradykinin levels due to ACE inhibition can lead to significant swelling, primarily because of its potent effects on endothelial cell function and histamine release. Thus, the interaction between these medications and histamine or bradykinin pathways can contribute to the development of angioedema [11]. In our case, the rapid onset of symptoms (within 20 min) following haloperidol administration suggests a histamine-mediated reaction, which is consistent with an immediate hypersensitivity response. Bradykinin-mediated angioedema, typically associated with ACE inhibitors, usually has a slower onset and is less likely in this context. The absence of prior exposure to ACE inhibitors and the rapid resolution with discontinuation of haloperidol further support a histamine-mediated mechanism. Haloperidol is a first-generation antipsychotic commonly used to manage acute psychosis and severe agitation. As a potent dopamine antagonist, it primarily targets the D2 receptors in the brain to help control symptoms associated with psychotic disorders. Despite its efficacy, haloperidol is associated with several side effects. The most common adverse effects include EPS such as tremors, rigidity, and bradykinesia, as well as akathisia. Oromandibular dystonia, a rare but notable side effect, involves involuntary muscle contractions affecting the mouth and jaw, leading to abnormal movements and postures. While oromandibular dystonia can be distressing, it is relatively uncommon compared to other EPS manifestations. Angioedema of the tongue, a serious and infrequent reaction, involves swelling of the tongue and can obstruct the airway, potentially leading to life-threatening situations. Although angioedema of the tongue is exceptionally rare, with only four documented cases in the literature linked to haloperidol, its severe nature warrants heightened awareness and prompt management [12]. Our case involves a 54-year-old male with a history of hepatitis B and late-onset schizophrenia who presented with acute tongue swelling shortly after taking two haloperidol pills. He had no known drug allergies and no recent epileptic or traumatic events. The swelling led to oromandibular dystonia, jaw clenching, and difficulties with speech and feeding, but he had no respiratory or cognitive issues. Clinical and imaging evaluations confirmed angioedema of the tongue without laryngeal involvement or trauma. He was admitted for monitoring, haloperidol was discontinued, and he was treated with antibiotics and enteral nutrition. The swelling and dystonia resolved by the third day, with complete recovery by the fifth day. The first case of tongue angioedema associated with haloperidol was reported in 2012 by Kahlon et al [13]. This case involved a 29-year-old male with schizophrenia who developed tongue swelling and protrusion after receiving a single intramuscular dose of haloperidol. The patient had no known allergies and had experienced similar swelling after a prior dose of haloperidol, which had resolved with diphenhydramine. The swelling recurred without further haloperidol administration, suggesting an allergic reaction rather than a cumulative drug effect. On examination, the patient's tongue and uvula were edematous, but he had no other symptoms such as fever or rash. He was treated with diphenhydramine and epinephrine, resulting in rapid improvement of the swelling. The patient was admitted for observation, and his condition resolved promptly with supportive care [13]. In contrast, our case involved a 54-year-old male who experienced acute tongue swelling 20 min after oral haloperidol, with additional symptoms of oromandibular dystonia and significant difficulties with speech and feeding. Unlike the swift resolution in Kahlon et al.'s [13] case, our patient required a more extended treatment period, including enteral feeding and antibiotics, with full recovery by the fifth day. The second case of tongue angioedema associated with haloperidol was reported in 2015 by Almadhyan [14]. This case involved a 47-year-old female with a history of diabetes mellitus, hypertension, and schizophrenia. She was on multiple medications, including haloperidol. The patient, who had a known allergy to Voltaren but no history of angioedema, was brought to the emergency department with agitation and restlessness. After administering midazolam twice without response, haloperidol was given intramuscularly. Within 10 min, she developed tongue swelling and protrusion. Initial treatment with diphenhydramine and epinephrine led to some symptom reduction, but the angioedema recurred severely and was resistant to further epinephrine. This worsening condition required tracheostomy due to compromised oxygen saturation. Following surgery and further observation, the patient's symptoms improved over the next few days, and she continued treatment for her other medical conditions [14]. While both cases illustrate severe angioedema linked to haloperidol, our patient's symptoms were less acute and did not necessitate invasive procedures. The third case of tongue angioedema associated with haloperidol, reported in 2017 by Masiran [15], involved a 27-year-old student with schizophrenia who experienced acute agitation and psychosis. After being administered haloperidol and midazolam, he developed painful tongue protrusion and swelling within hours. Despite initial supportive care and the discontinuation of haloperidol, his condition included involuntary tongue protrusion and mild edema, with no airway compromise. A detailed examination ruled out other conditions like meningitis and amphetamine intoxication. Treatment included haloperidol discontinuation, orphenadrine, dexamethasone, and benzhexol, leading to gradual improvement over several days. Compared to our case, which had a more straightforward resolution with conservative management, Masiran's [15] patient required an extended treatment regimen and was closely monitored for complications. The fourth case reports a similar presentation of dystonic reaction and swelling of the tongue following haloperidol administration in a 14-year-old male patient with recent psychosis confirmation. This case was also treated medically [16].

## 5. Conclusion

Angioedema is a localized subcutaneous and submucosal swelling evolving in deep tissue layers of areas like the face, lips, and throat. This condition can stem from various etiologies, mostly allergic reactions to foods, medications, or insect stings. Other acquired factors include nonallergic triggers such as certain medications like (ACE) inhibitors used for hypertension treatment. Angioedema can also be due to genetic factors with mutations causing deficit or dysfunction in the C1-inhibitor protein. Allergic mechanism of angioedema is typically immunoglobulin E-mediated causing increased vascular permeability, which results in a rapid onset of swelling. Haloperidol is a commonly prescribed antipsychotic medication for psychotic disorders, mainly schizophrenia. This drug presents a spectrum of adverse effects, primarily neurological. Its most frequent acute side effects include EPSs including dystonia and akathisia. Angioedema represents a rare but potentially life-threatening complication. Our case report highlights the infrequent occurrence of acute angioedema of the tongue following oral haloperidol administration, documented only in four reported instances. The rapid onset of tongue swelling in our patient necessitated immediate recognition and monitoring due to the risk of acute upper airway obstruction and respiratory compromise. This case underscores the importance of vigilance and prompt diagnosis in managing such rare adverse reactions associated with haloperidol.

## Data Availability

All data generated or analyzed during this study are included in this published article.

## Nomenclature

ACE: Angiotensin-converting enzyme
CRP: C-reactive protein
CT: Computed tomography
EPS: Extrapyramidal symptom.

## References

[1] Kazandjieva J., Christoff G. (2019). Angioedema as a Systemic Disease. *Clinics in Dermatology*.

[2] Lewis L. M. (2013). Angioedema: Etiology, Pathophysiology, Current and Emerging Therapies. *The Journal of Emergency Medicine*.

[3] Bühler L., Schmid B., Fabritius E., Grauvogel T. D. (2023). Das Angioödem in der Notaufnahme. *Medizinische Klinik–Intensivmedizin und Notfallmedizin*.

[4] Moellman J. J., Bernstein J. A., Lindsell C. (2014). A Consensus Parameter for the Evaluation and Management of Angioedema in the Emergency Department. *Academic Emergency Medicine*.

[5] Wedner H. J. (2020). Hereditary Angioedema: Pathophysiology (HAE Type I, HAE Type II, and HAE nC1-INH). *Allergy and Asthma Proceedings*.

[6] Proper S. P., Lavery W. J., Bernstein J. A. (2020). Definition and Classification of Hereditary Angioedema. *Allergy and Asthma Proceedings*.

[7] Patel G., Pongracic J. A. (2019). Hereditary and Acquired Angioedema. *Allergy and Asthma Proceedings*.

[8] Tam J. S. (2017). Cutaneous Manifestation of Food Allergy. *Immunology and Allergy Clinics of North America*.

[9] Müller U. (1989). Insektenstichallergie–Klinik, Diagnose und Therapie [Insect Sting Allergy--Clinical Aspects, Diagnosis and Therapy]. *Wiener Medizinische Wochenschrift (1946)*.

[10] James C., Bernstein J. A. (2017). Current and Future Therapies for the Treatment of Histamine-Induced Angioedema. *Expert Opinion on Pharmacotherapy*.

[11] Sinnathamby E. S., Urban B. T., Clark R. A. (2024). Etiology of Drug-Induced Edema: A Review of Dihydropyridine, Thiazolidinedione, and Other Medications Causing Edema. *Cureus*.

[12] Beach S. R., Gross A. F., Hartney K. E., Taylor J. B., Rundell J. R. (2020). Intravenous Haloperidol: A Systematic Review of Side Effects and Recommendations for Clinical Use. *General Hospital Psychiatry*.

[13] Kahlon S., Lee C., Chirurgi R., Worku Hassen G. (2012). Angioneurotic Edema Associated With Haloperidol. *Case Reports in Emergency Medicine*.

[14] AlMadhyan A. B. (2015). Angioedema Associated With Haloperidol. *International Journal of Health Sciences*.

[15] Masiran R. (2017). Persistent Ooromandibular Dystonia and Angioedema Secondary to Haloperidol. *BMJ Case Reports*.

[16] Strain A. (2022). Angioedema of the Tongue Due to Haloperidol. *The American Journal of Emergency Medicine*.

